# Challenges and prospects: women's education in contemporary Afghanistan

**DOI:** 10.3389/fgwh.2025.1477145

**Published:** 2025-03-20

**Authors:** Anonymous author 1, Anonymous author 2, Nasar Ahmad Shayan

**Affiliations:** 1 Department of Public Health and Infectious Diseases, Faculty of Medicine, Herat University, Herat, Afghanistan; 2 Department of Epidemiology and Biostatistics, Schulich School of Medicine and Dentistry, Western University, London, ON, Canada

**Keywords:** women, Afghanistan, Taliban, education, challenges

## Abstract

Since the fall of the republic government in Afghanistan on August 15, 2021, the situation for women's education has regressed drastically. This article explores the multifaceted impact of Afghanistan's Current DeFacto government policies on women's educational opportunities. With a historical overview of women's rights in Afghanistan, this article delves into the current restrictions imposed by the regime, including the ban on women's higher education and the limited scope of semi-higher education. This highlights the significant challenges faced by Afghan women, such as cultural barriers, economic hardships, and restrictive policies on women's rights. The article also discusses potential solutions, including international pressure, infrastructure development, and cultural shifts towards a more inclusive interpretation of Islam. By examining these factors, this article aims to provide a nuanced understanding of the ongoing struggle for women's rights and education in Afghanistan while emphasizing the resilience of Afghan women and the crucial role of global advocacy in supporting their fight for equality.

## Introduction

The fall of Kabul on August 15, 2021, marked a dramatic shift in Afghanistan's sociopolitical landscape, particularly affecting women's rights. This event led to the establishment of a De-facto government, which immediately imposed severe restrictions on women's freedom, including their rights to education. Historically, Afghan women have struggled to access education and have faced numerous cultural and political barriers. Under previous regimes, efforts were made to improve educational access and female participation in the public sphere. However, these gains were quickly reversed as the Taliban took control, reinstating a conservative interpretation of Islamic law that greatly restricted women's roles in society (1).

Since the the fall of the republic government, De-Facto government policies have directly affected women's access to education and employment. The De-facto government's declarations initially suggested a temporary ban on women's education, supposedly aligning the curriculum with Islamic principles. However, further statements and actions revealed a permanent intent to limit women's educational and occupational opportunities. Women are now forbidden from pursuing higher education, with exceptions in semi-higher health-related fields such as nursing and laboratory work. This ban has not only restricted educational access but also significantly impacted women's mental health and societal roles (2).

Moreover, the international community’s response has been mixed, with some nations and organizations attempting to pressure the De-Facto government to uphold women’s rights. However, the reality on the ground remains grim, with Afghan women facing increasing isolation and a loss of freedom. These actions have led to a dramatic decrease in the literacy rate among women, which currently stands at a mere 29.8%, one of the lowest in the world (3).

In contrast to existing studies, this article provides a comprehensive analysis of the current educational restrictions imposed since 2021. It highlights novel dimensions, including the use of underground networks and online platforms, and examines the role of religious leaders in framing inclusive Islamic interpretations to support education. Furthermore, the study updates the discourse by incorporating recent global advocacy efforts and presenting actionable strategies tailored to the evolving socio-political climate.

## Historical context

Afghanistan's history of women's education has been marked by significant fluctuations. During the reign of Amanullah Khan in the early 20th century, the country saw its first significant push towards modernization, including improvements in women's rights. However, this progress was met with strong resistance from conservative factions within Afghan society, leading to periodic regression. The Soviet invasion in 1979 and the subsequent civil war further destabilized the country, impacting all sectors, including education. Women suffered immensely, with many denying basic rights and access to education (4).

The rise of the Taliban in 1996 brought about a new era of repression. The regime imposed one of the most severe forms of Sharia law: banning women from public life, including work and education. This period saw complete exclusion of women from educational institutions and professional fields. After Taliban's ouster in 2001, Afghanistan experienced a period of relative freedom, with significant international support aimed at rebuilding its infrastructure and educational system. Women began to return to schools and universities, and their participation in the workforce increased. However, these gains are fragile and depend heavily on international support and security (5).

The post-2001 era also saw the establishment of legal frameworks to protect women's rights, including the 2004 Constitution, which enshrined gender equality and guaranteed women's rights to education and work. Despite these efforts, cultural and traditional barriers continue to limit the scope of such rights. The societal acceptance of women in education and the workforce remained limited, especially in rural areas where conservative values were deeply entrenched. The events of 2021 have undone much of the progress made, plunging the country back into an era of severe restrictions and gender segregation (6).

## Current situation

As of 2024, the situation for women in Afghanistan will be dire. The Current De-facto government has enforced a ban on women's higher education and has imposed strict dress codes and travel restrictions, effectively confining many women to their homes. The De-facto government's Ministry of Education has decreed that girls above sixth grade are not allowed to attend school, severely limiting their educational prospects. This policy has been justified under the guise of aligning the curriculum with Islamic values, yet it has effectively barred half of the population from accessing essential education (7).

The De-facto government allowed some women to continue working, primarily in the health and education sectors, but under stringent conditions. Women are required to adhere to strict dress codes and can only interact with their male colleagues under limited and controlled circumstances. These measures have not only restricted women's professional opportunities but have also perpetuated a climate of fear and uncertainty. Many women are forced to leave their jobs or work under humiliating and degrading conditions (8).

In response to these restrictions, some Afghan women have turned to underground educational networks and online platforms to continue their studies. Organizations such as LEARN have been instrumental in providing covert educational opportunities, although these initiatives are fraught with challenges, including limited Internet access and the constant threat of persecution by the De-facto government. Despite these efforts, the overall literacy rate among Afghan women remains alarmingly low, with few prospects for improvement under the current regime (9).

In response to the severe restrictions imposed by the Current De-facto government, numerous efforts have emerged in Afghanistan and beyond to support women's education. Notably, some education leaders have initiated private schools offering religious education to girls from grades 7 to 12, leveraging the fact that religious education is currently allowed for females. These institutions often frame formal and scientific education within a religious context to navigate the De-facto government's restrictions. International support has been instrumental in providing educational opportunities for Afghan girls, with many countries offering scholarships for both school and university education. Iranian scholarship has proven particularly favorable because it offers a unique advantage due to cultural and linguistic similarities, geographical proximity, and lower living costs. These efforts represent a beacon of hope for many Afghan women and girls striving to continue their education despite the oppressive environment.

## Challenges

The challenges faced by Afghan women in accessing education and employment are multi-faceted. One of the primary barriers is the ideological stance of the Current De-facto government, which enforces a strict interpretation of Sharia law that severely limits women's rights (10). This ideological framework is not only a political tool but also a means of maintaining control over the population. The De-facto government's use of religion to justify its policies has created an environment where questioning these rules can result in severe punishment, including imprisonment or worse (11).

Another significant challenge is the lack of infrastructure and resources for supporting women's education. Many schools and universities have been closed or repurposed, and there is a severe shortage of female teachers, especially in rural areas (12). This lack of infrastructure is compounded by economic hardships, as many families cannot afford to send their daughters to school even if opportunities are available. Economic instability and high levels of poverty exacerbate this situation, making education an unaffordable luxury for many Afghan families (13).

The third challenge lies in pervasive cultural and traditional norms that continue to restrict women's roles in society. Even before the fall of the republic government, many Afghan families were reluctant to send their daughters to school because of fear of social ostracism or physical harm (14). These cultural barriers are deeply rooted and often perpetuated by local religious leaders who support the De-facto government's strict policies. Overcoming these deeply entrenched beliefs requires a significant shift in societal attitudes, which is unlikely to occur under the current regime (2).

## Solutions

A multifaceted approach is required to address these challenges.

1. First, international pressure and diplomatic efforts must continue to hold the Current De-facto government accountable for human rights violations. Sanctions and other measures could be leveraged to compel the regime to allow women greater freedom. Additionally, international organizations and NGOs should work to provide support to underground education networks and online platforms, ensuring that Afghan women have access to alternative educational opportunities.
2. Second, there is a need to invest in infrastructure, particularly in rural areas. International aid can be directed towards building and refurbishing schools and universities, as well as training female teachers. This infrastructure development must be accompanied by economic support for families, such as scholarships and stipends, to encourage them to send their daughters to the school. Such measures can help alleviate the economic burden and make education more accessible.
3. Finally, changing cultural attitudes are crucial for the long-term improvement of women's rights in Afghanistan. This can be achieved through public awareness campaigns and educational programmes that promote gender equality and challenge traditional norms. Engaging local religious leaders in these efforts can also be beneficial as they have a significant influence on Afghan society. By promoting a more inclusive interpretation of Islamic teachings, leaders can help shift public opinion and support women's education and employment.

## Conclusion

The events of 2021 have brought unprecedented restrictions on women’s education in Afghanistan, reversing decades of progress and exacerbating societal inequalities. These restrictions have had far-reaching consequences, including a diminished healthcare workforce, rising poverty, and severe mental health challenges among Afghan women. Despite these challenges, Afghan women continue to exhibit remarkable resilience through underground education networks and online platforms. Addressing this crisis requires a multifaceted approach: international advocacy to pressure the regime, investments in infrastructure to improve access to education, and cultural shifts supported by local leaders to challenge discriminatory norms. By supporting Afghan women's right to education, the international community can help foster hope, stability, and progress in Afghanistan's future.

## Data Availability

The original contributions presented in the study are included in the article/Supplementary Material, further inquiries can be directed to the corresponding author.

## References

[1] Tan NF Ineli-Ciger M. Group-based protection of Afghan women and girls under the 1951 refugee convention. Int Comp Law Q. (2023) 72(3):793–817. 10.1017/S0020589323000283

[2] Skaine R. Women of Afghanistan in the Post-Taliban Era: How Lives Have Changed and Where They Stand Today. McFarland (2009). 10.5860/choice.46-4160

[3] Country Economy. Afghanistan - Literacy rate 2018. (2018). Available online at: https://countryeconomy.com/demography/literacy-rate/afghanistan (Accessed August 07, 2024).

[4] Kissane C. The way forward for girls’ education in Afghanistan. J Int Womens Stud. (2012) 13:10–28.

[5] Burridge N Maree Payne A Rahmani N. ‘Education is as important for me as water is to sustaining life’: perspectives on the higher education of women in Afghanistan. Gend Educ. (2016) 28(1):128–47. 10.1080/09540253.2015.1096922

[6] Rasekh Z Bauer H Manos M Iacopino V. Women’s health and human rights in Afghanistan. JAMA. (1998) 280(5):449–55. 10.1001/jama.280.5.449 9701081

[7] Omirzhanov Y Ghyasi M Bertayeva B. Taliban’s misconception of Islamic law in treatment with women rights. Saudi J Humanit Soc Sci. (2023) 8:122–31. 10.36348/sjhss.2023.v08i05.004

[8] Kayen HSS. Improvements and setbacks in women’s access to education: a case study of Afghanistan. Muslim Educ Rev. (2022) 1:19–36. 10.56529/mer.v1i1.5

[9] Sarhan N. ‘Because I am a girl’: identity and positioning in Afghani women narratives. Cairo Stud Engl. (2022). 10.21608/cse.2022.130478.1113 (cited August 7, 2024).

[10] Schulz JJ Schulz L. The darkest of ages: Afghan women under the taliban. Peace Confl J Peace Psychol. (1999) 5(3):237–54. 10.1207/s15327949pac0503_5

[11] Ramozi M Kaneda Y Barati H Ozaki A Mousavi SH. Earthquakes and Taliban decrees: the plight of Afghan women and children. Cureus. (2023) 15. 10.7759/cureus.48663 PMC10713278 38090400

[12] Palmer C. The Taliban’s war on women. Lancet. (1998) 352. 10.1016/S0140-6736(05)60848-3 9729009

[13] Kadir MAA Nurhaliza S. State responsibility of Afghanistan under Taliban regime. J Media Huk. (2023) 30:1–18. 10.18196/jmh.v30i1.16020

[14] Iacopino R. Education, a health imperative: the case of Afghanistan. Health Hum Rights. (1998) 3(2):98–108. 10343296

